# Prosthetic valve endocarditis secondary to *Tropheryma whipplei* in a patient with chronic polyarthritis

**DOI:** 10.1186/s13019-023-02287-1

**Published:** 2023-04-28

**Authors:** Mauricio J. Kahn, David M. Ennis, Dennis G. Delgado

**Affiliations:** Division of Infectious Diseases, University of Alabama at Birmingham, 1900 University Blvd, THT 229, Birmingham, AL 35294 USA

**Keywords:** Whipple’s endocarditis, Culture-negative endocarditis, Prosthetic valve endocarditis

## Abstract

**Background:**

Whipple’s disease is a chronic multisystemic infectious disease that rarely presents as culture-negative endocarditis. Most patients reported with *Tropheryma whipplei* endocarditis involve a native valve and few describe prosthetic valve disease.

**Case presentation:**

A patient with chronic polyarthritis and previous mitral valve replacement developed decompensated heart failure without fever. Transesophageal echocardiography revealed a prosthetic mitral valve vegetation and he underwent prosthetic mitral valve replacement. Blood and prosthetic mitral valve cultures were unrevealing. Broad-range polymerase chain reaction (PCR) of the extracted valve and subsequent Periodic-acid-Schiff (PAS) staining established the diagnosis of *T. whipplei* prosthetic valve endocarditis.

**Conclusion:**

Whipple’s disease may present as culture-negative infective endocarditis and affect prosthetic valves. Histopathology with PAS staining and broad-range PCR of excised valves are essential for the diagnosis. Greater clinical awareness and implementation of these diagnostic procedures should result in an increased reported incidence of this rare disease.

## Introduction

Patients with Whipple’s disease, a chronic multisystemic infection caused by the gram-positive bacillus *Tropheryma whipplei*, typically present with some combination of diarrhea, abdominal pain, weight loss, and polyarthralgias.[1, 2] Cardiac involvement, described in 20–55% of cases, may occur without gastrointestinal disease and most frequently presents as culture-negative infective endocarditis (CNIE).[3–5] Most patients with *T. whipplei* infective endocarditis (TWIE) have the disorder on a native valve and prosthetic valve endocarditis (PVE) is rarely reported.[1, 3, 5–11] In a recent case series, only 1 of 17 patients with TWIE had prosthetic valve involvement.[4] We present a patient with culture-negative PVE secondary to *T. whipplei*, diagnosed using molecular techniques.

## Case report

A 50-year-old man presented to our hospital with one month of dyspnea on exertion and lower extremity edema. Two weeks prior to admission, he was hospitalized at another institution with decompensated heart failure, severe mitral regurgitation, and possible pneumonia. He received diuretics and antibiotics and his symptoms improved. He was referred to our hospital to evaluate his mitral valvulopathy.

Past medical history included heart failure due to mitral valve regurgitation, atrial fibrillation, pulmonary thromboembolism, and inflammatory polyarthritis. Two years prior to admission, he underwent mitral valve revision complicated by recurrent mitral regurgitation and new tricuspid regurgitation requiring bioprosthetic mitral valve replacement and tricuspid valve repair. Histopathology of the extracted mitral valve revealed fibrosis, no evidence of endocarditis, and no other findings to suggest a specific diagnosis. Valvular tissue cultures were not obtained.

He complained of arthralgias that were controlled on colchicine and also epigastric abdominal pain. He denied fever, weight loss, or diarrhea. Other medications included carvedilol, furosemide, and warfarin. He had no medication allergies. There was no foreign travel, illicit drug, alcohol, or tobacco use. He was a retired welder.

Physical examination revealed a well-developed, well-nourished man who appeared fatigued in moderate respiratory distress. Temperature 37.3 °C, blood pressure 98/86 mmHg, heart rate 115, respiratory rate 25, and oxygen saturation 95% on 3 L of oxygen via nasal cannula. He had increased jugular venous distension, bilateral basilar crackles, grade III/VI holosystolic murmur in the mitral area, and bilateral lower extremity edema. There were no cutaneous stigmata of endocarditis. The remainder of his exam was normal.

Laboratory results revealed anemia and elevations of total bilirubin, alkaline phosphatase, and B-type natriuretic peptide. Computed tomography of the chest and abdomen revealed bilateral ground-glass opacities consistent with edema, a right pleural effusion, and mild mediastinal and retroperitoneal lymphadenopathy.

**Fig. 1:**
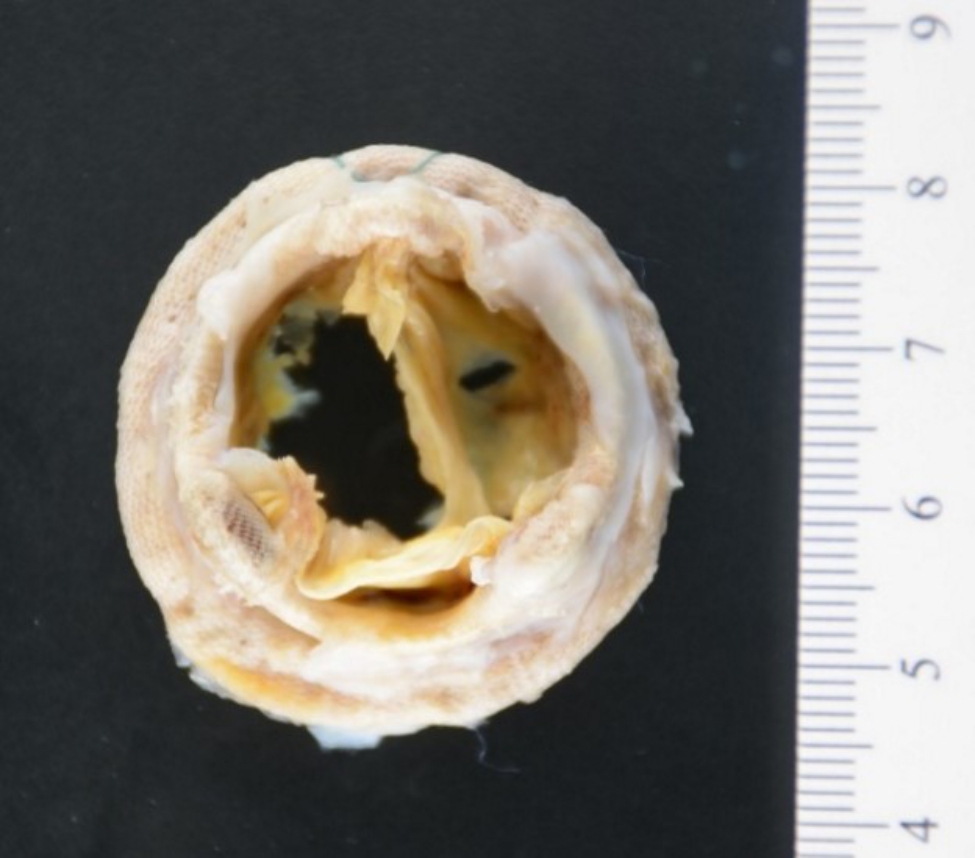
Extracted prosthetic mitral valve. Both leaflets appeared perforated. There were no vegetations or thrombi seen macroscopically. At the time of surgery, the infected valve annulus had been debrided. Scale marker in centimeters

He was admitted to the cardiac care unit and begun on furosemide and dobutamine infusions. A transthoracic echocardiogram revealed two small mobile echo-densities on the prosthetic mitral valve consistent with vegetations. On hospital day 3, admission blood cultures remained negative and transesophageal echocardiogram revealed a 1.1-centimeter mobile structure on the prosthetic mitral valve with severe regurgitation. Additional blood cultures were obtained and held for 14-days. Serologies for *Bartonella sp.* and *Coxiella burnetti* were negative. He was begun on intravenous vancomycin, gentamicin, and cefepime for culture-negative PVE. Cardiovascular surgery was consulted and since the patient was hemodynamically stable, they favored delaying surgery until blood cultures were negative at five days. He underwent a bioprosthetic mitral valve replacement on hospital day 9. The extracted valve (Fig. 1) was sent for cultures and broad-range PCR for bacterial, fungal, and mycobacterial organisms. Histopathology revealed vegetations with chronic inflammation.

Antibiotics were continued and he developed dialysis-dependent renal failure on hospital day 12. His antibiotics were transitioned to intravenous vancomycin, ceftriaxone, and ampicillin, and on hospital day 20 he was discharged on this regimen to complete a 6-week course. Blood and extracted valve cultures remained negative for microbial growth and he was maintained on hemodialysis.

**Fig. 2:**
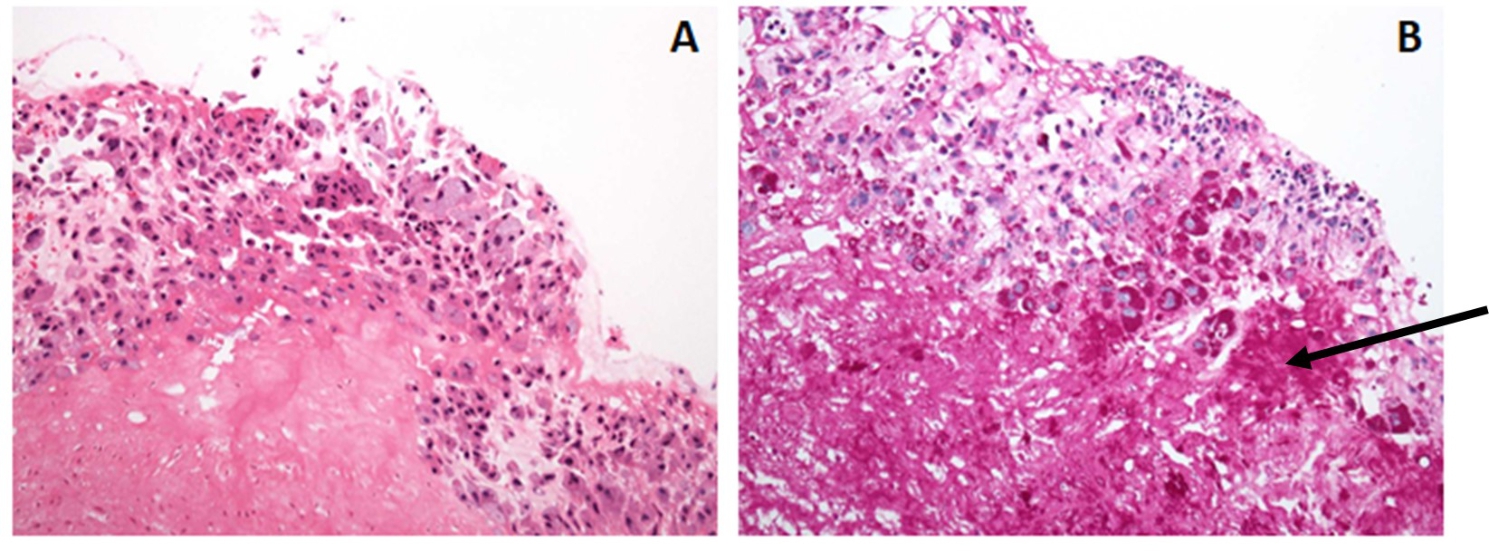
Prosthetic valve specimen. Hematoxylin and eosin stain (panel A). Granular periodic-acid-Schiff stain positive material consistent with *T. whipplei* organisms (panel B, see arrow). Both panels are at 20x magnification

On post-discharge day 5, broad-range PCR from the extracted prosthetic mitral valve returned positive for *Tropheryma whipplei*. In addition, Periodic-acid-Schiff (PAS) stain revealed macrophages containing granular PAS-positive material consistent with *T. whipplei* organisms (Fig. 2). Antibiotics were consolidated to monotherapy with intravenous ceftriaxone and after six weeks of therapy he improved with only mild residual dyspnea. After discussion with nephrology, he was transitioned to oral trimethoprim-sulfamethoxazole with plans to treat for at least twelve months.

## Discussion

Whipple’s disease (WD) is caused by *T. whipplei*, a ubiquitous environmental bacterium that has been detected in stool and saliva of healthy individuals and does not grow in blood cultures.[1, 4, 6] Although gastrointestinal manifestations are the most common presentation of WD, it may cause CNIE when it affects heart valves.[1] Because of diagnostic challenges, the exact incidence of endocarditis in patients with WD is unknown. CNIE represents approximately 5–20% of infective endocarditis cases.[12, 13] The incidence of *T. whipplei* in CNIE ranges from 2.6–6.3%.[2, 4, 11] In a series of 1135 patients undergoing valve replacements, 255 had bacterial endocarditis, 16 (6.3%) of which were secondary to *T. whipplei*.[2] Mortality in TWIE is approximately 24%, similar to other causes of bacterial endocarditis.[1, 4, 8].

Over 80% of TWIE cases occur in men, and the average age of presentation is 57.[1–4] Preexisting valvular disease is present in 21% of TWIE patients, and most involve the aortic (64%) and mitral (20%) valves.[1, 3, 4] Case series describe heart failure as the primary clinical manifestation in 76.5% of patients.[4] Our patient had a prosthetic mitral valve and presented with decompensated heart failure. Polyarthralgias, reported in 53% of TWIE cases, can precede the diagnosis by years and should raise suspicion for *T. whipplei* in patients with CNIE.[1, 4] Our patient had been followed by rheumatology for seven years with migratory inflammatory polyarthritis of undetermined etiology which was likely secondary to undiagnosed WD. Fever, reported in only 21% of patients with TWIE,[1] was absent in our patient.

The diagnosis of TWIE is challenging because in addition to negative blood cultures, typical features of infective endocarditis, including Duke’s criteria, are often absent.[1, 3] TWIE is diagnosed after valve excision using histopathology and broad-range PCR protocols that amplify *T. whipplei* DNA.[1, 3] Despite negative valve cultures, Periodic-acid-Schiff stain is positive in 83.4% of cases.[4] Therefore, it should be performed in all cases of CNIE. Submitting valvular tissue for broad-range PCR is also an important strategy in CNIE, and is particularly helpful for diagnosing TWIE in the absence of gastrointestinal symptoms.[6, 11] Dedicated PCR for *T. whipplei* has a higher sensitivity and should be considered if broad-range PCR is unrevealing.[8] Serum PCR is positive in 31.2% of patients with TWIE.[14].

*T. whipplei* infective endocarditis is usually treated with four weeks of intravenous ceftriaxone followed by oral trimethoprim-sulfamethoxazole or doxycycline and hydroxychloroquine for at least twelve months.[8] There is insufficient data to define the optimal antibiotic duration for TWIE affecting a prosthetic valve. However, based on the duration of parenteral antibiotics recommended for other bacterial causes of PVE, a 6-week course of intravenous ceftriaxone followed by a prolonged oral regimen as above seems prudent.

## Conclusion

*T. whipplei* infective endocarditis may present in the absence of gastrointestinal manifestations, occurs more frequently in middle-aged men, and presents as CNIE often without fever or stigmata of infective endocarditis. It may affect prosthetic valves, and chronic arthralgias should raise suspicion for the disease. Histopathology with PAS staining and broad-range PCR of excised valves are essential for the diagnosis. Greater clinical awareness and implementation of these diagnostic procedures should result in an increased reported incidence and appropriate management of patients with *T. whipplei* infective endocarditis.

## Data Availability

Data sharing is not applicable to this article as no datasets were generated or analyzed during the current study.

## List of Abbreviations

PCR: Polymerase chain reaction
PAS: Periodic-acid-Schiff
CNIE: Culture-negative infective endocarditis
TWIE: *Tropheryma whipplei* infective endocarditis
PVE: Prosthetic valve endocarditis
WD: Whipple’s disease
